# Diaqua­bis(5-carb­oxy-2-propyl-1*H*-imidazole-4-carboxyl­ato-κ^2^ *N* ^3^,*O* ^4^)magnesium(II) 3.5-hydrate

**DOI:** 10.1107/S1600536810005684

**Published:** 2010-02-17

**Authors:** Xiang-Yun Liu, Li-Hua Liu

**Affiliations:** a Department of Chemistry and Chemical Engineering, Henan University of Urban Construction, Pingdingshan, Henan 467044, People’s Republic of China

## Abstract

In the title complex, [Mg(C_8_H_9_N_2_O_4_)_2_(H_2_O)_2_]·3.5H_2_O, the Mg^II^ atom is six-coordinated by two *N*,*O*-bidentate 5-carb­oxy-2-propyl-1*H*-imidazole-4-carboxyl­ate ligands and two water mol­ecules, forming a distorted octa­hedral environment. The complex mol­ecules are linked into a three-dimensional network by N—H⋯O and O—H⋯O hydrogen-bonding inter­actions. The propyl groups are disordered over two sites, with site occupancies of 0.755 (7):0.245 (7) and 0.556 (13):0.444 (13).

## Related literature

For related structures, see: Sengupta *et al.* (2001 ▶); Song *et al.* (2010 ▶); Wang *et al.* (2004 ▶); Yan *et al.* (2010 ▶).
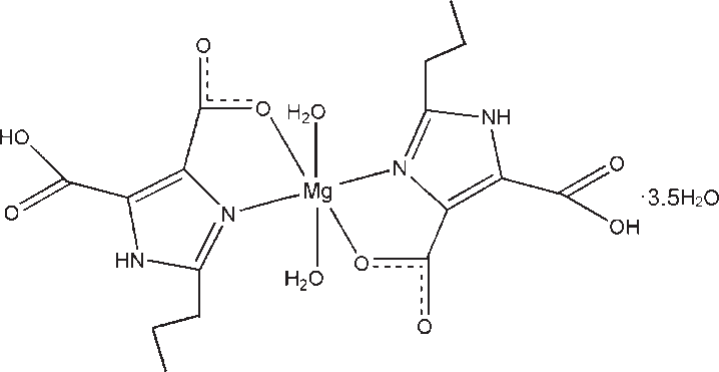


## Experimental

### 

#### Crystal data

[Mg(C_8_H_9_N_2_O_4_)_2_(H_2_O)_2_]·3.5H_2_O
*M* *_r_* = 517.74
Triclinic, 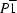

*a* = 10.516 (1) Å
*b* = 10.5332 (11) Å
*c* = 11.3989 (13) Å
α = 83.288 (1)°
β = 81.783 (1)°
γ = 86.458 (2)°
*V* = 1239.8 (2) Å^3^
*Z* = 2
Mo *K*α radiation
μ = 0.14 mm^−1^
*T* = 298 K
0.48 × 0.38 × 0.35 mm

#### Data collection

Bruker SMART 1000 CCD diffractometer
Absorption correction: multi-scan (*SADABS*; Sheldrick, 1996 ▶) *T*_min_ = 0.935, *T*_max_ = 0.952
6478 measured reflections
4321 independent reflections
2780 reflections with *I* > 2σ(*I*)
*R*_int_ = 0.028

#### Refinement

*R*[*F* ^2^ > 2σ(*F* ^2^)] = 0.058
*wR*(*F* ^2^) = 0.178
*S* = 1.00
4321 reflections
378 parameters
H-atom parameters constrained
Δρ_max_ = 0.58 e Å^−3^
Δρ_min_ = −0.31 e Å^−3^

### 

Data collection: *SMART* (Bruker, 2007 ▶); cell refinement: *SAINT* (Bruker, 2007 ▶); data reduction: *SAINT*; program(s) used to solve structure: *SHELXS97* (Sheldrick, 2008 ▶); program(s) used to refine structure: *SHELXL97* (Sheldrick, 2008 ▶); molecular graphics: *SHELXTL* (Sheldrick, 2008 ▶) and *DIAMOND* (Brandenburg, 1999 ▶); software used to prepare material for publication: *SHELXTL*.

## Supplementary Material

Crystal structure: contains datablocks I, 1R. DOI: 10.1107/S1600536810005684/hy2281sup1.cif

Structure factors: contains datablocks I. DOI: 10.1107/S1600536810005684/hy2281Isup2.hkl

Additional supplementary materials:  crystallographic information; 3D view; checkCIF report

## Figures and Tables

**Table 1:** Hydrogen-bond geometry (Å, °)

*D*—H⋯*A*	*D*—H	H⋯*A*	*D*⋯*A*	*D*—H⋯*A*
N2—H2⋯O12	0.86	1.89	2.745 (4)	170
N4—H4⋯O13	0.86	1.91	2.737 (4)	162
O3—H3⋯O2	0.82	1.70	2.511 (3)	173
O7—H7⋯O6	0.82	1.65	2.461 (3)	172
O9—H9*C*⋯O8^i^	0.85	1.88	2.732 (3)	177
O9—H9*D*⋯O11^ii^	0.85	1.83	2.678 (4)	176
O10—H10*C*⋯O4^iii^	0.85	1.94	2.787 (3)	174
O10—H10*D*⋯O8^iv^	0.85	2.06	2.905 (3)	174
O11—H11*C*⋯O2^v^	0.85	1.95	2.794 (3)	172
O11—H11*D*⋯O5^vi^	0.85	2.05	2.893 (3)	172
O12—H12*C*⋯O7^vi^	0.85	2.05	2.888 (4)	167
O12—H12*D*⋯O14^v^	0.85	1.84	2.672 (6)	167
O13—H13*C*⋯O14	0.85	1.85	2.643 (6)	156
O13—H13*D*⋯O11^vii^	0.85	2.07	2.869 (5)	156
O14—H14*G*⋯O1^viii^	0.85	2.00	2.816 (5)	162
O14—H14*H*⋯O1^ix^	0.85	1.98	2.799 (5)	162

Symmetry codes: (i) 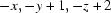; (ii) 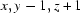; (iii) 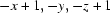; (iv) 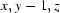; (v) 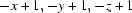; (vi) 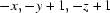; (vii) 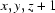; (viii) 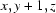; (ix) 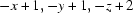.

**Fig. 1.**
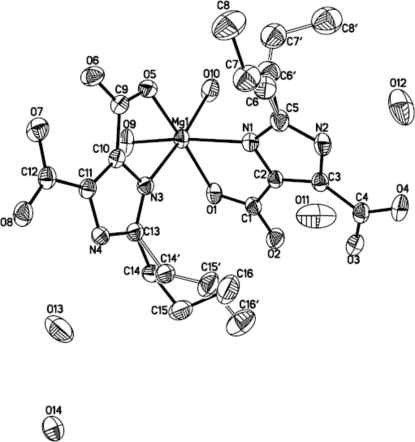
Molecular structure of the title compound. Displacement ellipsoids are drawn at the 30% probability level. H atoms are omitted for clarity.

**Fig. 2.**
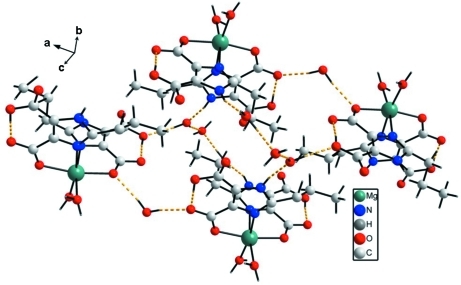
A view of the three-dimensional network constructed by N—H···O and O—H···O hydrogen bonding interactions (dashed lines).

## supplementary crystallographic information

### Comment

Imidazole-4,5-dicarboxylate ligands with efficient N,*O*-donors have been been widely used to obtain new complexes with excellent properties (Sengupta *et al.*, 2001; Song *et al.*,2010; Wang *et al.*, 2004; Yan *et al.*, 2010). Bearing this in mind, we introduced Mg(CH_3_CO_2_)_2_ and 2-propyl-1*H*-imidazole-4,5-dicarboxylic acid into reaction so as to obtain a new Mg^II^ complex.

As illustrated in Fig. 1, the title complex molecule contains one Mg^II^ ion, two mono-deprotonated 2-propyl-1*H*-imidazole-4,5-dicarboxylate ligands, two coordinated water molecules and three and half uncoordinated water molecules. The Mg ^II^ atom is six-coordinated by two N,*O*-bidentate ligands and two water molecules in a slightly distorted octahedral environment. Both ligands coordinate through N atoms and carboxylate O atoms in a bidentate chelate fashion, forming two five-membered Mg, O, C, C, N rings. Uncoordinated solvent water molecules are located in cavities of the three-dimensional network, participating in N—H···O and O—H···O hydrogen bonds, which contribute to the stability of the network. The two propyl residues are disordered over two sites, with site occupancies of 0.755 (7):0.245 (7) and 0.556 (13):0.444 (13).

### Experimental

The title compound was prepared by the hydrothermal reaction of Mg(CH_3_CO_2_)_2_ (0.5 mmol, 0.07 g) and 2-propyl-1*H*-imidazole-4,5-dicarboxylic acid (0.5 mmol, 0.99 g) in 15 ml of H_2_O solution. The reaction was performed in a Teflon-lined autoclave (20 ml), which was heated at 433 K for 2 d. Crystals of the title compound were obtained by slow evaporation of the solvent at room temperature.

### Refinement

C- and N-bound H atoms were placed at calculated positions and were treated as riding atoms, with C—H = 0.97 (CH_2_) and 0.96 (CH_3_) Å and N—H = 0.86 Å and with *U*_iso_(H) = 1.2(1.5 for methyl)*U*_eq_(C,*N*). The water H atoms were located in a difference map and refined as riding, with O—H = 0.85 Å and *U*_iso_(H) = 1.2*U*_eq_(O). H atoms of carboxyl groups were located in a difference map and refined as riding, with O—H = 0.82 Å and *U*_iso_(H) = 1.5*U*_eq_(O).

### Crystal data

**Table:**

[Mg(C_8_H_9_N_2_O_4_)_2_(H_2_O)_2_]·3.5H_2_O	*Z* = 2
*M**_r_* = 517.74	*F*(000) = 546
Triclinic, *P*1	*D*_x_ = 1.387 Mg m^−^^3^
Hall symbol: -P 1	Mo *K*α radiation, λ = 0.71073 Å
*a* = 10.516 (1) Å	Cell parameters from 2051 reflections
*b* = 10.5332 (11) Å	θ = 2.5–23.9°
*c* = 11.3989 (13) Å	µ = 0.14 mm^−^^1^
α = 83.288 (1)°	*T* = 298 K
β = 81.783 (1)°	Block, colorless
γ = 86.458 (2)°	0.48 × 0.38 × 0.35 mm
*V* = 1239.8 (2) Å^3^	

### Data collection

**Table:**

Bruker SMART 1000 CCD diffractometer	4321 independent reflections
Radiation source: fine-focus sealed tube	2780 reflections with *I* > 2σ(*I*)
graphite	*R*_int_ = 0.028
φ and ω scans	θ_max_ = 25.0°, θ_min_ = 1.8°
Absorption correction: multi-scan (*SADABS*; Sheldrick, 1996)	*h* = −11→12
*T*_min_ = 0.935, *T*_max_ = 0.952	*k* = −7→12
6478 measured reflections	*l* = −12→13

### Refinement

**Table:**

Refinement on *F*^2^	Primary atom site location: structure-invariant direct methods
Least-squares matrix: full	Secondary atom site location: difference Fourier map
*R*[*F*^2^ > 2σ(*F*^2^)] = 0.058	Hydrogen site location: inferred from neighbouring sites
*wR*(*F*^2^) = 0.178	H-atom parameters constrained
*S* = 1.00	*w* = 1/[σ^2^(*F*_o_^2^) + (0.0865*P*)^2^ + 0.6255*P*] where *P* = (*F*_o_^2^ + 2*F*_c_^2^)/3
4321 reflections	(Δ/σ)_max_ = 0.001
378 parameters	Δρ_max_ = 0.58 e Å^−^^3^
0 restraints	Δρ_min_ = −0.31 e Å^−^^3^

### Fractional atomic coordinates and isotropic or equivalent isotropic displacement parameters (Å^2^)

**Table:**

	*x*	*y*	*z*	*U*_iso_*/*U*_eq_	Occ. (<1)
Mg1	0.20602 (10)	0.14342 (10)	0.81297 (9)	0.0417 (3)	
N1	0.2821 (2)	0.1678 (3)	0.6227 (2)	0.0429 (6)	
N2	0.3556 (3)	0.1891 (3)	0.4316 (2)	0.0518 (7)	
H2	0.3565	0.2023	0.3556	0.062*	
N3	0.2165 (2)	0.3506 (2)	0.8307 (2)	0.0406 (6)	
N4	0.2228 (2)	0.5498 (3)	0.8701 (2)	0.0455 (7)	
H4	0.2525	0.6192	0.8859	0.055*	
O1	0.4034 (2)	0.1060 (2)	0.81403 (18)	0.0481 (6)	
O2	0.5972 (2)	0.1009 (3)	0.7049 (2)	0.0601 (7)	
O3	0.6855 (2)	0.1268 (3)	0.4872 (2)	0.0639 (7)	
H3	0.6563	0.1120	0.5578	0.096*	
O4	0.6100 (2)	0.1666 (2)	0.3161 (2)	0.0652 (7)	
O5	0.0167 (2)	0.2175 (2)	0.7964 (2)	0.0483 (6)	
O6	−0.1219 (2)	0.3840 (2)	0.8141 (2)	0.0602 (7)	
O7	−0.1173 (2)	0.6067 (2)	0.8584 (2)	0.0588 (7)	
H7	−0.1178	0.5304	0.8504	0.088*	
O8	0.0222 (2)	0.7397 (2)	0.8968 (2)	0.0580 (7)	
O9	0.1655 (3)	0.1075 (2)	0.9919 (2)	0.0653 (8)	
H9C	0.1071	0.1529	1.0290	0.078*	
H9D	0.1832	0.0418	1.0385	0.078*	
O10	0.1704 (3)	−0.0415 (2)	0.7915 (2)	0.0674 (8)	
H10C	0.2394	−0.0744	0.7569	0.081*	
H10D	0.1228	−0.1018	0.8241	0.081*	
O11	0.2291 (3)	0.8974 (3)	0.1314 (3)	0.0858 (10)	
H11C	0.2762	0.8940	0.1864	0.103*	
H11D	0.1592	0.8619	0.1597	0.103*	
O12	0.3299 (3)	0.2189 (4)	0.1936 (3)	0.1094 (13)	
H12C	0.2705	0.2676	0.1671	0.131*	
H12D	0.3729	0.1839	0.1359	0.131*	
O13	0.3434 (3)	0.7359 (3)	0.9551 (3)	0.1053 (12)	
H13C	0.4103	0.7795	0.9419	0.126*	
H13D	0.2914	0.7684	1.0088	0.126*	
O14	0.5130 (5)	0.9089 (5)	0.9622 (4)	0.0584 (13)	0.50
H14G	0.4816	0.9784	0.9298	0.070*	0.50
H14H	0.5333	0.9220	1.0292	0.070*	0.50
C1	0.4763 (3)	0.1168 (3)	0.7168 (3)	0.0438 (8)	
C2	0.4130 (3)	0.1509 (3)	0.6087 (3)	0.0382 (7)	
C3	0.4614 (3)	0.1646 (3)	0.4896 (3)	0.0430 (8)	
C4	0.5913 (3)	0.1533 (3)	0.4242 (3)	0.0482 (8)	
C5	0.2496 (3)	0.1891 (4)	0.5137 (3)	0.0563 (10)	
C6	0.1138 (11)	0.1962 (10)	0.4864 (9)	0.064 (2)	0.755 (7)
H6A	0.0598	0.1492	0.5511	0.077*	0.755 (7)
H6B	0.1108	0.1566	0.4142	0.077*	0.755 (7)
C7	0.0631 (7)	0.3333 (8)	0.4701 (6)	0.082 (2)	0.755 (7)
H7A	0.1103	0.3778	0.3995	0.099*	0.755 (7)
H7B	0.0753	0.3760	0.5383	0.099*	0.755 (7)
C8	−0.0804 (6)	0.3375 (8)	0.4573 (7)	0.111 (3)	0.755 (7)
H8A	−0.0927	0.2905	0.3929	0.166*	0.755 (7)
H8B	−0.1106	0.4248	0.4410	0.166*	0.755 (7)
H8C	−0.1278	0.2999	0.5301	0.166*	0.755 (7)
C6'	0.129 (3)	0.259 (3)	0.478 (3)	0.061 (7)	0.245 (7)
H6'1	0.1463	0.3122	0.4029	0.073*	0.245 (7)
H6'2	0.0875	0.3102	0.5389	0.073*	0.245 (7)
C7'	0.047 (2)	0.145 (2)	0.4661 (19)	0.081 (6)	0.245 (7)
H7'1	0.0451	0.0872	0.5389	0.097*	0.245 (7)
H7'2	−0.0401	0.1781	0.4606	0.097*	0.245 (7)
C8'	0.091 (2)	0.069 (2)	0.361 (2)	0.106 (8)	0.245 (7)
H8'1	0.0665	0.1152	0.2899	0.158*	0.245 (7)
H8'2	0.0511	−0.0122	0.3755	0.158*	0.245 (7)
H8'3	0.1827	0.0549	0.3526	0.158*	0.245 (7)
C9	−0.0087 (3)	0.3316 (3)	0.8131 (3)	0.0440 (8)	
C10	0.0964 (3)	0.4090 (3)	0.8322 (3)	0.0394 (7)	
C11	0.0983 (3)	0.5332 (3)	0.8570 (3)	0.0414 (7)	
C12	−0.0035 (3)	0.6349 (3)	0.8720 (3)	0.0458 (8)	
C13	0.2919 (3)	0.4388 (3)	0.8542 (3)	0.0461 (8)	
C14	0.4291 (18)	0.4147 (17)	0.8779 (12)	0.053 (3)	0.556 (13)
H14A	0.4549	0.3255	0.8714	0.064*	0.556 (13)
H14B	0.4359	0.4324	0.9584	0.064*	0.556 (13)
C15	0.5185 (8)	0.5000 (8)	0.7884 (11)	0.072 (3)	0.556 (13)
H15A	0.4907	0.5889	0.7943	0.086*	0.556 (13)
H15B	0.6050	0.4883	0.8094	0.086*	0.556 (13)
C16	0.5214 (18)	0.472 (2)	0.6607 (18)	0.099 (7)	0.556 (13)
H16A	0.5381	0.3817	0.6561	0.149*	0.556 (13)
H16B	0.5879	0.5184	0.6104	0.149*	0.556 (13)
H16C	0.4398	0.4973	0.6346	0.149*	0.556 (13)
C14'	0.434 (2)	0.435 (2)	0.8282 (14)	0.055 (4)	0.444 (13)
H14C	0.4671	0.3481	0.8467	0.066*	0.444 (13)
H14D	0.4674	0.4882	0.8799	0.066*	0.444 (13)
C15'	0.484 (2)	0.481 (2)	0.6993 (19)	0.073 (6)	0.444 (13)
H15C	0.4440	0.4348	0.6467	0.087*	0.444 (13)
H15D	0.4614	0.5711	0.6831	0.087*	0.444 (13)
C16'	0.6296 (14)	0.4600 (12)	0.6737 (12)	0.097 (5)	0.444 (13)
H16D	0.6694	0.4989	0.7304	0.146*	0.444 (13)
H16E	0.6594	0.4979	0.5946	0.146*	0.444 (13)
H16F	0.6517	0.3699	0.6799	0.146*	0.444 (13)

### Atomic displacement parameters (Å^2^)

**Table:**

	*U*^11^	*U*^22^	*U*^33^	*U*^12^	*U*^13^	*U*^23^
Mg1	0.0463 (6)	0.0375 (6)	0.0393 (6)	0.0013 (5)	0.0014 (5)	−0.0057 (5)
N1	0.0440 (16)	0.0449 (16)	0.0392 (15)	0.0004 (13)	−0.0037 (12)	−0.0055 (12)
N2	0.0598 (18)	0.0601 (19)	0.0335 (14)	0.0063 (15)	−0.0045 (13)	−0.0041 (13)
N3	0.0397 (15)	0.0359 (15)	0.0455 (15)	0.0011 (12)	−0.0020 (11)	−0.0077 (12)
N4	0.0455 (16)	0.0369 (15)	0.0544 (17)	−0.0044 (13)	0.0001 (12)	−0.0136 (13)
O1	0.0561 (14)	0.0522 (14)	0.0338 (12)	0.0090 (11)	−0.0037 (10)	−0.0042 (10)
O2	0.0467 (15)	0.0847 (19)	0.0490 (14)	0.0051 (13)	−0.0072 (11)	−0.0112 (13)
O3	0.0505 (15)	0.081 (2)	0.0572 (15)	−0.0037 (14)	0.0082 (12)	−0.0142 (15)
O4	0.0756 (18)	0.0645 (17)	0.0461 (15)	0.0060 (14)	0.0160 (12)	−0.0019 (12)
O5	0.0453 (13)	0.0449 (14)	0.0563 (14)	−0.0062 (11)	−0.0051 (10)	−0.0118 (11)
O6	0.0408 (14)	0.0581 (16)	0.0834 (18)	0.0030 (12)	−0.0140 (12)	−0.0103 (14)
O7	0.0511 (15)	0.0474 (15)	0.0758 (17)	0.0120 (12)	−0.0069 (12)	−0.0071 (14)
O8	0.0629 (16)	0.0412 (14)	0.0641 (16)	0.0011 (12)	0.0120 (12)	−0.0083 (12)
O9	0.0830 (18)	0.0568 (16)	0.0445 (14)	0.0250 (14)	0.0133 (12)	0.0008 (12)
O10	0.0755 (18)	0.0428 (15)	0.0765 (17)	−0.0136 (13)	0.0259 (14)	−0.0151 (13)
O11	0.087 (2)	0.078 (2)	0.098 (2)	−0.0325 (17)	−0.0461 (17)	0.0259 (17)
O12	0.131 (3)	0.138 (3)	0.0634 (19)	0.048 (2)	−0.0415 (19)	−0.021 (2)
O13	0.113 (3)	0.090 (3)	0.129 (3)	0.003 (2)	−0.041 (2)	−0.056 (2)
O14	0.072 (3)	0.060 (3)	0.045 (3)	0.006 (3)	−0.017 (2)	−0.006 (2)
C1	0.047 (2)	0.0414 (19)	0.0438 (19)	0.0021 (15)	−0.0063 (15)	−0.0119 (15)
C2	0.0417 (18)	0.0346 (17)	0.0378 (17)	0.0005 (14)	−0.0015 (13)	−0.0072 (13)
C3	0.0495 (19)	0.0372 (18)	0.0415 (18)	−0.0001 (15)	−0.0029 (15)	−0.0064 (14)
C4	0.056 (2)	0.0393 (19)	0.046 (2)	−0.0010 (16)	0.0065 (17)	−0.0071 (15)
C5	0.055 (2)	0.071 (3)	0.044 (2)	0.0056 (19)	−0.0095 (17)	−0.0099 (18)
C6	0.063 (5)	0.076 (7)	0.058 (4)	−0.007 (6)	−0.018 (3)	−0.006 (5)
C7	0.072 (4)	0.091 (5)	0.083 (4)	0.003 (4)	−0.017 (3)	0.003 (4)
C8	0.068 (4)	0.132 (7)	0.126 (6)	0.010 (4)	−0.023 (4)	0.013 (5)
C6'	0.060 (15)	0.064 (18)	0.057 (12)	0.005 (18)	−0.014 (10)	−0.002 (14)
C7'	0.072 (14)	0.095 (17)	0.075 (13)	−0.001 (12)	−0.015 (11)	−0.001 (12)
C8'	0.092 (15)	0.117 (19)	0.108 (17)	−0.004 (14)	−0.025 (13)	0.001 (15)
C9	0.0417 (19)	0.045 (2)	0.0439 (18)	−0.0009 (16)	−0.0028 (14)	−0.0023 (15)
C10	0.0393 (17)	0.0366 (18)	0.0393 (17)	0.0017 (14)	0.0013 (13)	−0.0020 (14)
C11	0.0438 (18)	0.0407 (19)	0.0368 (17)	0.0000 (15)	0.0004 (13)	−0.0009 (14)
C12	0.055 (2)	0.041 (2)	0.0374 (18)	0.0005 (16)	0.0043 (15)	0.0007 (15)
C13	0.0423 (18)	0.0410 (19)	0.055 (2)	−0.0019 (15)	0.0004 (15)	−0.0118 (16)
C14	0.045 (5)	0.055 (7)	0.062 (8)	−0.005 (4)	−0.010 (7)	−0.015 (7)
C15	0.050 (5)	0.064 (5)	0.099 (8)	−0.012 (4)	0.002 (5)	−0.015 (5)
C16	0.091 (19)	0.097 (10)	0.098 (13)	−0.009 (10)	0.031 (9)	−0.015 (8)
C14'	0.048 (6)	0.049 (8)	0.068 (11)	−0.004 (6)	0.000 (10)	−0.016 (9)
C15'	0.051 (10)	0.070 (8)	0.089 (17)	0.006 (7)	0.017 (10)	−0.015 (10)
C16'	0.060 (9)	0.090 (9)	0.133 (11)	0.005 (6)	0.003 (7)	0.003 (7)

### Geometric parameters (Å, °)

**Table:**

Mg1—O9	2.019 (2)	C5—C6'	1.51 (3)
Mg1—O10	2.055 (3)	C6—C7	1.508 (11)
Mg1—O1	2.090 (2)	C6—H6A	0.9700
Mg1—O5	2.118 (2)	C6—H6B	0.9700
Mg1—N1	2.193 (3)	C7—C8	1.535 (9)
Mg1—N3	2.226 (3)	C7—H7A	0.9700
N1—C5	1.325 (4)	C7—H7B	0.9700
N1—C2	1.365 (4)	C8—H8A	0.9600
N2—C5	1.349 (4)	C8—H8B	0.9600
N2—C3	1.370 (4)	C8—H8C	0.9600
N2—H2	0.8600	C6'—C7'	1.54 (3)
N3—C13	1.332 (4)	C6'—H6'1	0.9700
N3—C10	1.369 (4)	C6'—H6'2	0.9700
N4—C13	1.354 (4)	C7'—C8'	1.52 (3)
N4—C11	1.363 (4)	C7'—H7'1	0.9700
N4—H4	0.8600	C7'—H7'2	0.9700
O1—C1	1.252 (4)	C8'—H8'1	0.9600
O2—C1	1.262 (4)	C8'—H8'2	0.9600
O3—C4	1.303 (4)	C8'—H8'3	0.9600
O3—H3	0.8200	C9—C10	1.467 (5)
O4—C4	1.213 (4)	C10—C11	1.372 (4)
O5—C9	1.246 (4)	C11—C12	1.474 (5)
O6—C9	1.280 (4)	C13—C14'	1.48 (2)
O7—C12	1.285 (4)	C13—C14	1.506 (19)
O7—H7	0.8200	C14—C15	1.533 (17)
O8—C12	1.227 (4)	C14—H14A	0.9700
O9—H9C	0.8500	C14—H14B	0.9700
O9—H9D	0.8500	C15—C16	1.52 (2)
O10—H10C	0.8500	C15—H15A	0.9700
O10—H10D	0.8500	C15—H15B	0.9700
O11—H11C	0.8501	C16—H16A	0.9600
O11—H11D	0.8500	C16—H16B	0.9600
O12—H12C	0.8499	C16—H16C	0.9600
O12—H12D	0.8499	C14'—C15'	1.52 (2)
O13—H13C	0.8500	C14'—H14C	0.9700
O13—H13D	0.8499	C14'—H14D	0.9700
O14—H14G	0.8500	C15'—C16'	1.52 (2)
O14—H14H	0.8500	C15'—H15C	0.9700
C1—C2	1.481 (4)	C15'—H15D	0.9700
C2—C3	1.374 (4)	C16'—H16D	0.9600
C3—C4	1.465 (5)	C16'—H16E	0.9600
C5—C6	1.500 (11)	C16'—H16F	0.9600
			
O9—Mg1—O10	91.24 (11)	C8—C7—H7B	109.7
O9—Mg1—O1	93.24 (10)	H7A—C7—H7B	108.2
O10—Mg1—O1	94.26 (11)	C5—C6'—C7'	101.1 (19)
O9—Mg1—O5	92.42 (10)	C5—C6'—H6'1	111.6
O10—Mg1—O5	95.35 (11)	C7'—C6'—H6'1	111.6
O1—Mg1—O5	168.73 (10)	C5—C6'—H6'2	111.6
O9—Mg1—N1	170.33 (11)	C7'—C6'—H6'2	111.6
O10—Mg1—N1	87.42 (10)	H6'1—C6'—H6'2	109.4
O1—Mg1—N1	77.33 (9)	C8'—C7'—C6'	117 (2)
O5—Mg1—N1	97.24 (10)	C8'—C7'—H7'1	108.1
O9—Mg1—N3	89.86 (10)	C6'—C7'—H7'1	108.1
O10—Mg1—N3	171.92 (12)	C8'—C7'—H7'2	108.1
O1—Mg1—N3	93.67 (10)	C6'—C7'—H7'2	108.1
O5—Mg1—N3	76.60 (9)	H7'1—C7'—H7'2	107.3
N1—Mg1—N3	92.82 (10)	C7'—C8'—H8'1	109.5
C5—N1—C2	106.2 (3)	C7'—C8'—H8'2	109.5
C5—N1—Mg1	144.1 (2)	H8'1—C8'—H8'2	109.5
C2—N1—Mg1	109.73 (19)	C7'—C8'—H8'3	109.5
C5—N2—C3	108.5 (3)	H8'1—C8'—H8'3	109.5
C5—N2—H2	125.7	H8'2—C8'—H8'3	109.5
C3—N2—H2	125.7	O5—C9—O6	123.0 (3)
C13—N3—C10	105.7 (3)	O5—C9—C10	118.2 (3)
C13—N3—Mg1	144.0 (2)	O6—C9—C10	118.8 (3)
C10—N3—Mg1	109.6 (2)	N3—C10—C11	110.4 (3)
C13—N4—C11	108.7 (3)	N3—C10—C9	117.6 (3)
C13—N4—H4	125.6	C11—C10—C9	131.9 (3)
C11—N4—H4	125.6	N4—C11—C10	105.0 (3)
C1—O1—Mg1	118.6 (2)	N4—C11—C12	122.6 (3)
C4—O3—H3	109.5	C10—C11—C12	132.3 (3)
C9—O5—Mg1	117.5 (2)	O8—C12—O7	123.4 (3)
C12—O7—H7	109.5	O8—C12—C11	120.1 (3)
Mg1—O9—H9C	119.2	O7—C12—C11	116.5 (3)
Mg1—O9—H9D	130.5	N3—C13—N4	110.1 (3)
H9C—O9—H9D	108.5	N3—C13—C14'	125.5 (9)
Mg1—O10—H10C	107.2	N4—C13—C14'	121.5 (9)
Mg1—O10—H10D	140.1	N3—C13—C14	125.3 (7)
H10C—O10—H10D	108.2	N4—C13—C14	124.0 (7)
H11C—O11—H11D	108.6	C13—C14—C15	110.3 (9)
H12C—O12—H12D	108.7	C13—C14—H14A	109.6
H13C—O13—H13D	107.8	C15—C14—H14A	109.6
H14G—O14—H14H	108.8	C13—C14—H14B	109.6
O1—C1—O2	125.2 (3)	C15—C14—H14B	109.6
O1—C1—C2	116.1 (3)	H14A—C14—H14B	108.1
O2—C1—C2	118.6 (3)	C16—C15—C14	113.1 (11)
N1—C2—C3	110.1 (3)	C16—C15—H15A	109.0
N1—C2—C1	118.1 (3)	C14—C15—H15A	109.0
C3—C2—C1	131.7 (3)	C16—C15—H15B	109.0
N2—C3—C2	104.8 (3)	C14—C15—H15B	109.0
N2—C3—C4	121.6 (3)	H15A—C15—H15B	107.8
C2—C3—C4	133.5 (3)	C13—C14'—C15'	113.6 (16)
O4—C4—O3	121.5 (3)	C13—C14'—H14C	108.9
O4—C4—C3	121.4 (3)	C15'—C14'—H14C	108.9
O3—C4—C3	117.1 (3)	C13—C14'—H14D	108.9
N1—C5—N2	110.3 (3)	C15'—C14'—H14D	108.9
N1—C5—C6	124.3 (5)	H14C—C14'—H14D	107.7
N2—C5—C6	125.0 (5)	C16'—C15'—C14'	111 (2)
N1—C5—C6'	126.9 (12)	C16'—C15'—H15C	109.3
N2—C5—C6'	118.1 (12)	C14'—C15'—H15C	109.3
C5—C6—C7	110.8 (7)	C16'—C15'—H15D	109.3
C5—C6—H6A	109.5	C14'—C15'—H15D	109.3
C7—C6—H6A	109.5	H15C—C15'—H15D	108.0
C5—C6—H6B	109.5	C15'—C16'—H16D	109.5
C7—C6—H6B	109.5	C15'—C16'—H16E	109.5
H6A—C6—H6B	108.1	H16D—C16'—H16E	109.5
C6—C7—C8	109.9 (6)	C15'—C16'—H16F	109.5
C6—C7—H7A	109.7	H16D—C16'—H16F	109.5
C8—C7—H7A	109.7	H16E—C16'—H16F	109.5
C6—C7—H7B	109.7		
			
O10—Mg1—N1—C5	79.7 (4)	C2—N1—C5—C6'	−156.2 (14)
O1—Mg1—N1—C5	174.6 (4)	Mg1—N1—C5—C6'	26.8 (15)
O5—Mg1—N1—C5	−15.4 (4)	C3—N2—C5—N1	1.0 (4)
N3—Mg1—N1—C5	−92.2 (4)	C3—N2—C5—C6	−172.1 (6)
O10—Mg1—N1—C2	−97.2 (2)	C3—N2—C5—C6'	158.3 (13)
O1—Mg1—N1—C2	−2.28 (19)	N1—C5—C6—C7	97.0 (7)
O5—Mg1—N1—C2	167.7 (2)	N2—C5—C6—C7	−90.8 (8)
N3—Mg1—N1—C2	90.9 (2)	C6'—C5—C6—C7	−8(3)
O9—Mg1—N3—C13	81.6 (4)	C5—C6—C7—C8	−173.2 (6)
O1—Mg1—N3—C13	−11.7 (4)	N1—C5—C6'—C7'	−100.8 (18)
O5—Mg1—N3—C13	174.1 (4)	N2—C5—C6'—C7'	106.1 (17)
N1—Mg1—N3—C13	−89.1 (4)	C6—C5—C6'—C7'	−6.8 (19)
O9—Mg1—N3—C10	−87.2 (2)	C5—C6'—C7'—C8'	−70 (2)
O1—Mg1—N3—C10	179.55 (19)	Mg1—O5—C9—O6	−174.7 (2)
O5—Mg1—N3—C10	5.30 (19)	Mg1—O5—C9—C10	5.3 (4)
N1—Mg1—N3—C10	102.1 (2)	C13—N3—C10—C11	−0.2 (3)
O9—Mg1—O1—C1	−179.6 (2)	Mg1—N3—C10—C11	173.0 (2)
O10—Mg1—O1—C1	88.9 (2)	C13—N3—C10—C9	−177.8 (3)
O5—Mg1—O1—C1	−59.6 (6)	Mg1—N3—C10—C9	−4.6 (3)
N1—Mg1—O1—C1	2.5 (2)	O5—C9—C10—N3	−0.1 (4)
N3—Mg1—O1—C1	−89.5 (2)	O6—C9—C10—N3	180.0 (3)
O9—Mg1—O5—C9	83.4 (2)	O5—C9—C10—C11	−177.1 (3)
O10—Mg1—O5—C9	174.8 (2)	O6—C9—C10—C11	2.9 (5)
O1—Mg1—O5—C9	−36.7 (6)	C13—N4—C11—C10	−0.3 (3)
N1—Mg1—O5—C9	−97.1 (2)	C13—N4—C11—C12	178.3 (3)
N3—Mg1—O5—C9	−5.9 (2)	N3—C10—C11—N4	0.3 (3)
Mg1—O1—C1—O2	177.7 (3)	C9—C10—C11—N4	177.5 (3)
Mg1—O1—C1—C2	−2.2 (4)	N3—C10—C11—C12	−178.1 (3)
C5—N1—C2—C3	1.2 (4)	C9—C10—C11—C12	−0.9 (6)
Mg1—N1—C2—C3	179.3 (2)	N4—C11—C12—O8	0.3 (5)
C5—N1—C2—C1	−176.1 (3)	C10—C11—C12—O8	178.4 (3)
Mg1—N1—C2—C1	2.0 (3)	N4—C11—C12—O7	−179.3 (3)
O1—C1—C2—N1	0.0 (4)	C10—C11—C12—O7	−1.1 (5)
O2—C1—C2—N1	180.0 (3)	C10—N3—C13—N4	0.0 (4)
O1—C1—C2—C3	−176.7 (3)	Mg1—N3—C13—N4	−169.0 (3)
O2—C1—C2—C3	3.4 (5)	C10—N3—C13—C14'	−161.0 (8)
C5—N2—C3—C2	−0.2 (4)	Mg1—N3—C13—C14'	29.9 (9)
C5—N2—C3—C4	177.5 (3)	C10—N3—C13—C14	171.5 (7)
N1—C2—C3—N2	−0.6 (3)	Mg1—N3—C13—C14	2.5 (8)
C1—C2—C3—N2	176.2 (3)	C11—N4—C13—N3	0.2 (4)
N1—C2—C3—C4	−178.0 (3)	C11—N4—C13—C14'	162.1 (8)
C1—C2—C3—C4	−1.1 (6)	C11—N4—C13—C14	−171.5 (7)
N2—C3—C4—O4	0.9 (5)	N3—C13—C14—C15	122.3 (10)
C2—C3—C4—O4	177.9 (3)	N4—C13—C14—C15	−67.3 (11)
N2—C3—C4—O3	−178.4 (3)	C14'—C13—C14—C15	24 (3)
C2—C3—C4—O3	−1.4 (6)	C13—C14—C15—C16	−63.5 (15)
C2—N1—C5—N2	−1.4 (4)	N3—C13—C14'—C15'	81.0 (19)
Mg1—N1—C5—N2	−178.4 (3)	N4—C13—C14'—C15'	−78.0 (18)
C2—N1—C5—C6	171.8 (6)	C14—C13—C14'—C15'	179 (5)
Mg1—N1—C5—C6	−5.2 (8)	C13—C14'—C15'—C16'	−173.1 (15)

### Hydrogen-bond geometry (Å, °)

**Table:**

*D*—H···*A*	*D*—H	H···*A*	*D*···*A*	*D*—H···*A*
N2—H2···O12	0.86	1.89	2.745 (4)	170
N4—H4···O13	0.86	1.91	2.737 (4)	162
O3—H3···O2	0.82	1.70	2.511 (3)	173
O7—H7···O6	0.82	1.65	2.461 (3)	172
O9—H9C···O8^i^	0.85	1.88	2.732 (3)	177
O9—H9D···O11^ii^	0.85	1.83	2.678 (4)	176
O10—H10C···O4^iii^	0.85	1.94	2.787 (3)	174
O10—H10D···O8^iv^	0.85	2.06	2.905 (3)	174
O11—H11C···O2^v^	0.85	1.95	2.794 (3)	172
O11—H11D···O5^vi^	0.85	2.05	2.893 (3)	172
O12—H12C···O7^vi^	0.85	2.05	2.888 (4)	167
O12—H12D···O14^v^	0.85	1.84	2.672 (6)	167
O13—H13C···O14	0.85	1.85	2.643 (6)	156
O13—H13D···O11^vii^	0.85	2.07	2.869 (5)	156
O14—H14G···O1^viii^	0.85	2.00	2.816 (5)	162
O14—H14H···O1^ix^	0.85	1.98	2.799 (5)	162

Symmetry codes: (i) −*x*, −*y*+1, −*z*+2; (ii) *x*, *y*−1, *z*+1; (iii) −*x*+1, −*y*, −*z*+1; (iv) *x*, *y*−1, *z*; (v) −*x*+1, −*y*+1, −*z*+1; (vi) −*x*, −*y*+1, −*z*+1; (vii) *x*, *y*, *z*+1; (viii) *x*, *y*+1, *z*; (ix) −*x*+1, −*y*+1, −*z*+2.
